# Magnetic fibrin nanofiber hydrogel delivering iron oxide magnetic nanoparticles promotes peripheral nerve regeneration

**DOI:** 10.1093/rb/rbae075

**Published:** 2024-06-20

**Authors:** Juncong Hong, Dongze Wu, Haitao Wang, Zhe Gong, Xinxin Zhu, Fang Chen, Zihang Wang, Mingchen Zhang, Xiumei Wang, Xiangqian Fang, Shuhui Yang, Jinjin Zhu

**Affiliations:** Department of Orthopaedic Surgery, Sir Run Shaw Hospital, Zhejiang University School of Medicine & Key Laboratory of Musculoskeletal System Degeneration and Regeneration Translational Research of Zhejiang, Hangzhou, Zhejiang 310016, China; Department of Anesthesiology, The First People’s Hospital of Linping District, Hangzhou, Zhejiang 311100, China; Department of Spinal Surgery, The First Affiliated Hospital of Ningbo University, Ningbo, Zhejiang 315000, China; Department of Orthopaedic Surgery, Sir Run Shaw Hospital, Zhejiang University School of Medicine & Key Laboratory of Musculoskeletal System Degeneration and Regeneration Translational Research of Zhejiang, Hangzhou, Zhejiang 310016, China; Department of Orthopaedic Surgery, Sir Run Shaw Hospital, Zhejiang University School of Medicine & Key Laboratory of Musculoskeletal System Degeneration and Regeneration Translational Research of Zhejiang, Hangzhou, Zhejiang 310016, China; Stomatology Hospital, School of Stomatology, Zhejiang University School of Medicine, Zhejiang Provincial Clinical Research Center for Oral Diseases, Key Laboratory of Oral Biomedical Research of Zhejiang Province, Cancer Center of Zhejiang University, Engineering Research Center of Oral Biomaterials and Devices of Zhejiang Province, Hangzhou, Zhejiang 310000, China; School of Materials Science and Engineering, Zhejiang-Mauritius Joint Research Center for Biomaterials and Tissue Engineering, Zhejiang Sci-Tech University, Hangzhou, Zhejiang 310018, China; School of Materials Science and Engineering, Zhejiang-Mauritius Joint Research Center for Biomaterials and Tissue Engineering, Zhejiang Sci-Tech University, Hangzhou, Zhejiang 310018, China; School of Materials Science and Engineering, Zhejiang-Mauritius Joint Research Center for Biomaterials and Tissue Engineering, Zhejiang Sci-Tech University, Hangzhou, Zhejiang 310018, China; State Key Laboratory of New Ceramics and Fine Processing, Key Laboratory of Advanced Materials, School of Materials Science and Engineering, Tsinghua University, Beijing 100084, China; Department of Orthopaedic Surgery, Sir Run Shaw Hospital, Zhejiang University School of Medicine & Key Laboratory of Musculoskeletal System Degeneration and Regeneration Translational Research of Zhejiang, Hangzhou, Zhejiang 310016, China; School of Materials Science and Engineering, Zhejiang-Mauritius Joint Research Center for Biomaterials and Tissue Engineering, Zhejiang Sci-Tech University, Hangzhou, Zhejiang 310018, China; Department of Orthopaedic Surgery, Sir Run Shaw Hospital, Zhejiang University School of Medicine & Key Laboratory of Musculoskeletal System Degeneration and Regeneration Translational Research of Zhejiang, Hangzhou, Zhejiang 310016, China

**Keywords:** peripheral nerve regeneration, fibrin, iron oxide magnetic nanoparticles, hydrogel

## Abstract

Peripheral nerve injury is a debilitating condition that have a profound impact on the overall quality of an individual’s life. The repair of peripheral nerve defects continues to present significant challenges in the field. Iron oxide magnetic nanoparticles (IONPs) have been recognized as potent nanotools for promoting the regeneration of peripheral nerves due to their capability as biological carriers and their ability to template the hydrogel structure under an external magnetic field. This research used a fibrin nanofiber hydrogel loaded with IONPs (IONPs/fibrin) to promote the regeneration of peripheral nerves in rats. *In vitro* examination of PC12 cells on various concentrations of IONPs/fibrin hydrogels revealed a remarkable increase in NGF and VEGF expression at 2% IONPs concentration. The biocompatibility and degradation of 2% IONPs/fibrin hydrogel were assessed using the *in vivo* imaging system, demonstrating subcutaneous degradation within a week without immediate inflammation. Bridging a 10-mm sciatic nerve gap in Sprague Dawley rats with 2% IONPs/fibrin hydrogel led to satisfactory morphological recovery of myelinated nerve fibers. And motor functional recovery in the 2% IONPs/fibrin group was comparable to autografts at 6, 9 and 12 weeks postoperatively. Hence, the composite fibrin hydrogel incorporating 2% IONPs exhibits potential for peripheral nerve regeneration.

## Graphical abstract

**Figure:**
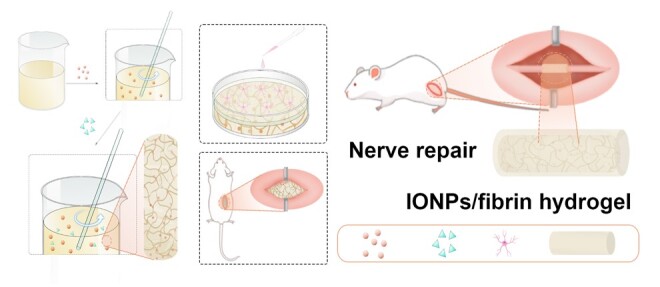

## Introduction

Every year, around 300 000 peripheral nerve injuries (PNIs) take place in Europe, while there are approximately 200 000 PNIs in the United States annually. These injuries are frequently observed in patients who have experienced trauma [1]. Due to its elevated socioeconomic status, the United States annually allocates approximately $150 billion towards PNIs, a figure that directly corresponds to the considerable socioeconomic impact [2, 3]. The treatment of peripheral nerve injury is usually based on the severity of the injury. Nerve injuries are classified into three broad categories depending on the severity of the injury: neuropraxia, axonotmesis and neurotmesis. For neurotmesis repair, current methods include direct end to end repair, grafts and synthetic nerve conduits. The electromagnetic stimulation is usually used for postoperative rehabilitation or functional recovery of neuropraxia. In clinical practice, autologous nerve transplantation is considered the gold standard for treating large-gap peripheral nerve injuries. Nevertheless, its widespread use is hindered by limitations in donor availability and the potential complications at the donor site [4]. Researchers have investigated nerve guidance conduits as potential alternatives to nerve autografts in peripheral nerve regeneration. The primary focus in peripheral nerve regeneration is currently on the development of innovative nerve guidance conduits, which involves various techniques such as conduit fillers, cellular approaches, growth factors and electromagnetic stimulation, individually or in combination [5]. However, the current obstacles in peripheral nerve repair revolve around various factors including: slow axonal regeneration (1 mm/day); long nerve regeneration distances; the improper reconnection of nerve axons; inadequate blood supply during nerve regeneration; inhibitory signals within the extracellular matrix; and a deficiency of the required Schwann cells to populate the nerve injury site [6, 7]. Hence, the quest to create an effective method to nerve regeneration remains a key area of study in the field of repairing damaged peripheral nerves.

Magnetic stimulation, harnessing the power of magnetic nanoparticles along with magnetic fields, is regarded as a potential approach for healing tissue injuries. Magnetic scaffolds are created by incorporating magnetic nanoparticles into biocompatible polymers, such as fibrin, collagen, gelatin and hyaluronic acid [8–11]. By introducing magnetic nanoparticles, the characteristics of the scaffold biomaterials are altered, and they acquire magnetic properties. Utilizing stationary magnetic fields can assist in creating magnetic support structures that have different properties in different directions, whereas fluctuating magnetic fields provide the ability to remotely activate substances and cells [12]. The proliferation and differentiation of various cells such as mesenchymal stem cells (MSCs) and dental pulp stem cells can be enhanced by magnetic scaffolds, according to reports [8, 13–15]. Moreover, the utilization of magnetic scaffolds has been demonstrated to promote the growth of new blood vessels, carrying advantageous potential for the restoration of tissues [16, 17]. Transition metal oxides give rise to iron oxide nanoparticles (IONPs), which are widely used as magnetic nanoparticles for various applications including templating hydrogels, aiding in targeted transport, and inducing mechanical tension [18–20]. The researchers discovered that IONPs, when subjected to mechanical tension of around 1pN, were able to successfully promote the growth of PC12 cell extensions called neurites [21, 22]. In addition to PC12 cells, Gao *et al.* discovered that using chondroitinase ABC/polyethylenimine functionalized IONPs in magnetofection notably boosts the migration of Schwann cells within the magnetic field’s axis [23]. In the field of neuroscience, magnetic scaffolds and magnetic stimulation have garnered increasing attention due to their potential in promoting the growth of neurites [20, 24, 25].

The use of suitable biomaterials in the encapsulation of IONPs is extremely vital. Certain biomaterials have inherent traits that include the ability to seamlessly integrate with living tissue, break down naturally over time, and effectively encapsulate IONPs. Fibrin hydrogel distinguishes itself as a highly promising biomaterial utilized for promoting the regrowth of peripheral nerves. The fibrin hydrogel possesses a high level of elasticity and negative compressibility, and its elastic modulus closely resembles that of the extracellular matrix (ECM) in nerves [26–28]. Multiple research papers have documented its benefits in the regrowth of peripheral nerves [29, 30]. Furthermore, the fibrin hydrogel exhibits the ability to attract macrophages that counteract inflammation, enhance the production of anti-inflammatory cytokines, and hinder the secretion of inflammatory substances [31]. However, there is still limited understanding regarding the impact of IONPs/fibrin hydrogel on the regeneration of peripheral nerves and their potential neurotoxic effects.

**Figure 1.**
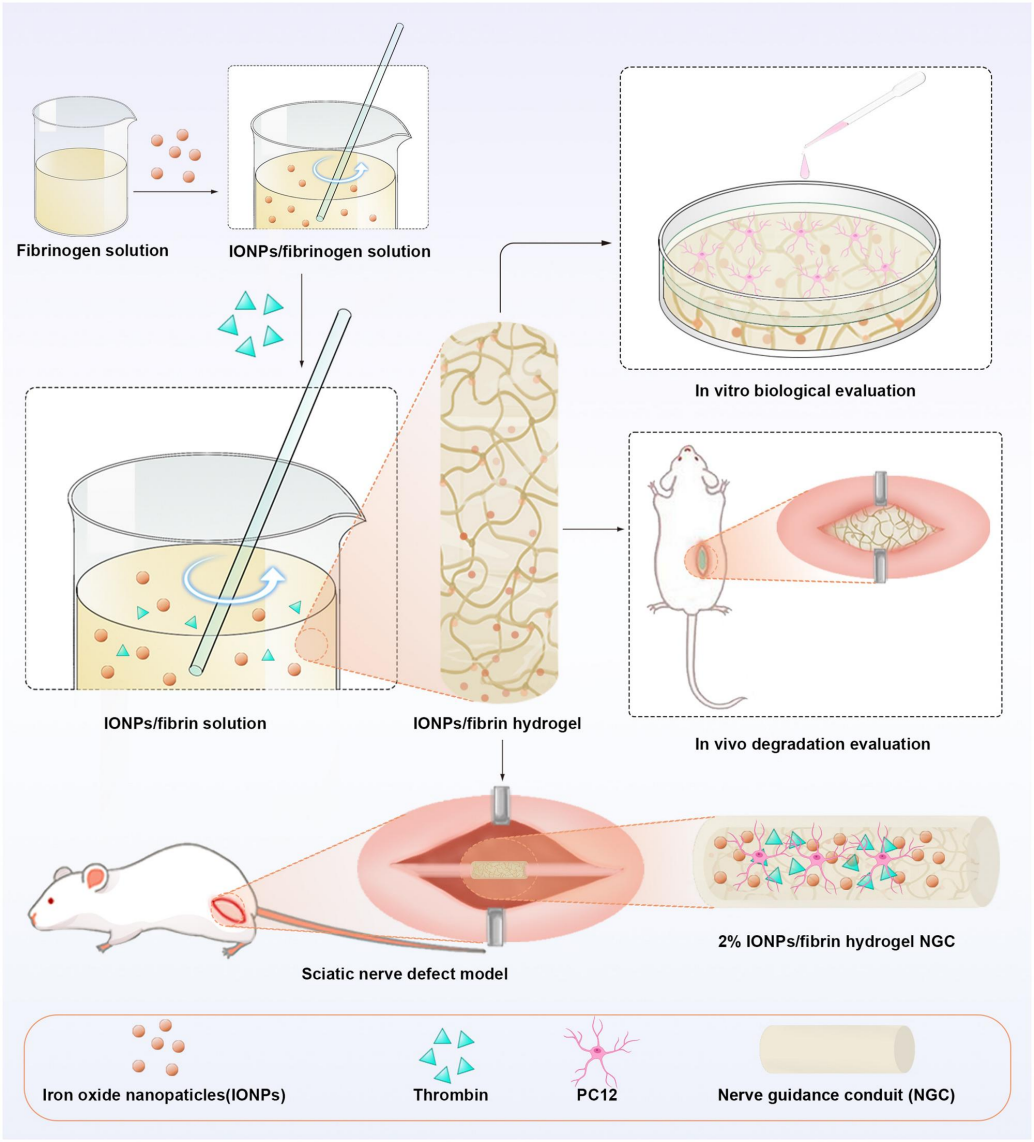
The IONPs/fibrin hydrogel’s fabrication procedure, *in vitro* biocompatibility evaluation, and *in vivo* degradation evaluation are illustrated in the diagram. The composite hydrogel was preloaded into a chitosan tube to connect a 10-mm gap in the sciatic nerve of rats.

The objective of this study was to assess the biocompatibility, *in vivo* degradation, and the application of IONPs/fibrin hydrogel implants in sciatic nerve defect (Figure 1). The scanning electron microscopy (SEM) and energy dispersive X-ray spectroscopy (EDS) techniques were used to analyze the microscopic structure and elemental composition of the IONPs/fibrin hydrogels. The rheological measurements were performed to evaluate the viscoelasticity properties of the IONPs/fibrin hydrogels. Cell adhesion, viability and proliferation were evaluated using the Cell counting kit 8 (CCK8) assay, as well as live-dead cell staining, actin filament staining and the cell viability assay. The integrity of the cell membrane was assessed though the use of a lactate dehydrogenase release assay. The experiment effectively showcased alterations in the gene and protein expressions of PC12 cells when exposed to varying concentrations of IONPs, as well as when utilizing a 2% IONPs/fibrin hydrogel composite. Furthermore, the degradation and tolerance of the materials were analyzed by subcutaneously implanting 2% IONPs/fibrin hydrogel into the rats. Ultimately, a 10-mm-long gap in the sciatic nerve of rats was bridged using a hollow chitosan nerve conduit filled with a mixture of 2% IONPs/fibrin hydrogel. And the morphological features of the regenerated myelinated axons and the restoration of motor function were evaluated.

## Materials and methods

### Preparation of hydrogels and nerve graft

IONPs were acquired from Shanghai So-Fe Biomedicine Co. Ltd, China. The fibrin hydrogel was fabricated by directly mixing 20 mg/ml fibrinogen solution (Sigma-Aldrich, USA) with 20 U/ml thrombin solution (Sigma-Aldrich, USA) in a volume ratio of 10:1. The different concentrations of IONPs (1%, 2%, 5%, 10% equal with 10, 20, 50 and 100 μg/ml) were previously added to the fibrinogen solution to fabricate the IONPs/fibrin hydrogels with different concentrations of IONPs. The glacial acetic acid used in the experiment was obtained from Sinopharm Chemical Reagent Co. Ltd, China. Chitosan was acquired from Shanghai Aladdin Bio-Chem Technology Co. Ltd in China. 6% chitosan solution was produced by dissolving chitosan powder in 2% acetic acid solution. After that, a 2-mm diameter glass rod was utilized as a representation to fabricate a nerve guide conduit having an internal diameter of 2 mm and a thickness of 0.2 mm. To neutralize acetic acid, the chitosan nerve conduit that was created underwent immersion in a solution of 2% sodium hydroxide. Subsequently, the neutralized chitosan conduit was divided into sections measuring 10 mm and submerged in a solution of 75% ethanol. As a prerequisite to implantation, the chitosan conduit underwent cleansing using a sterilized PBS solution, followed by injection of either fibrin hydrogels or IONPs/fibrin hydrogels.

### Transmission electron microscopy analysis

The morphology of IONPs is characterized by transmission electron microscopy (TEM).

### Rheological properties

Rheological properties of the hydrogels were measured using a rheometer at room temperature (25°C). Stress/strain sweeps (0.1—1000% at 1 Hz) were performed. Each experiment was performed in triplicate.

### Scanning electron microscopy analysis

The samples were immobilized using 2.5% glutaraldehyde for 2 h, followed by washing in PBS and dehydration in 50%, 70%, 80%, 90%, 95%, 100% ethanol solution for 30 min. Subsequently, the CO_2_ critical point dryer (Tousimis, USA) was used in order to dry the hydrogel samples. The samples were coated with a layer of platinum, and then observed using a field emission SEM (Thermo Fisher Scientific, USA) at an accelerating voltage of 5 kV. An SEM equipped with an EDS was utilized for chemical element analysis when operating at an acceleration voltage of 15 kV. ImageJ software accurately enabled the precise determination of the porosity and fiber diameter of various samples.

### Cell culture

PC12 cells (rat pheochromocytoma 12) obtained from Procell in China were cultured in RPMI 1640 medium, which was supplemented with 10% horse serum (HS) from Meilunbio, China, along with 5% fetal bovine serum (FBS) and 1% penicillin-streptomycin (PS) provided by Meilunbio, China. The cells were incubated in a humidified incubator set at a constant temperature of 37°C with 95% air and 5% CO_2_ to facilitate their optimal growth and proliferation, with the culture medium being refreshed every 2 days.

### Calcein AM/propidium iodide staining

The live/dead assay (Meilunbio, China) was performed using calcein AM (live)/propidium iodide (PI) (dead). PC12 cells were placed onto fibrin hydrogels with different concentrations of IONPs (0, 10, 20 and 50 μg/ml) at a density of 1 × 10^5^ in a confocal dish. After 1, 3 and 5 days, cells were incubated with calcein AM/PI solution at 37°C for 30 min, and observed using a confocal laser scanning microscope (Leica, Germany). The percentage of live and dead cells was analyzed using ImageJ software.

### Cell viability assay

The Luminescent Cell Viability Assay (Vazyme Biotech, China) was employed to assess the cell viability. PC12 cells were seeded on fibrin hydrogels with varying concentrations of IONPs (0, 10, 20, 50 and 100 μg/ml) at a density of 1 × 10^4^ cells per well in 48-well plates. After 24 h, the cell culture medium was aspirated and washed with PBS. Then, working solution was added in a 1:1 ratio and allowed to incubate at 37°C for 30 min, followed by analyzing using UV spectrophotometry to determine the extent of enzymatic activity. In order to assess the absorbance at 480 nm, the contents of each well, consisting of 100 μl of a reactive solution, were transferred to a fresh 96-well plate.

### Cell counting kit 8 assays

To assess cell proliferation in this study, PC12 cells were placed onto the surface of fibrin hydrogels with different concentrations of IONPs (0, 10, 20, 50 and 100 μg/ml) at a density of 1 × 10^4^ cells per well in 48-well plates. After being cultured for 1, 3 and 5 days, PC12 cells were rinsed three times using PBS. Following that, 300 μl of fresh medium was combined with 30 μl CCK-8 reagent in each well, and the plate was incubated for 2 h at 37°C with 5% CO_2_. Subsequently, the optical density (OD) value was recorded at 450 nm using a microplate reader after transferring 100 μl of the reactive solution from each well to a new 96-well plate for measurement.

### LDH releasing assay

The PC12 cells were introduced onto the hydrogel samples in a 48-well plate with a cell density of 5 × 10^3^ cells/well and left to cultivate for 48 h. Subsequently, the samples were rinsed with a PBS aqueous solution. The culture medium was replaced with serum-free medium, with 150 μl used in each well. The culture plate was segregated into various groups, including a negative control, hydrogel samples and a positive control. LDH release solution (15 μl) was introduced into each well of the positive control group and incubated for 1 h at 37°C. The 48-well plate was spun at 400 g at 4°C for 5 min. A volume of 120 μl of the supernatant was carefully aspirated and added in a new 96-well plate. After adding 60 μl LDH detection working solution (Beyotime, China), the plate was shaken slowly for 30 min. At this point, the absorbance was recorded at 490 nm and the cytotoxicity of each sample was calculated according to the formula: cytotoxicity (%) = (hydrogel samples group-negative control group)/(positive control group-negative control group) × 100.

### PC12 cell adhesion

The PC12 cells were cultured on hydrogels with varying concentrations of IONPs for 1 and 3 days. Briefly, the cells were fixed with 4% paraformaldehyde, permeabilized with 0.1% Triton X-100, and incubated with 1% bovine serum albumin (BSA). Actin filaments were stained using CoraLite Plus 488-Phalloidin (Proteintech, China), while nuclei were stained with 4′,6-diamidino-2-phenylindole (DAPI, Solarbio, China). The cells were imaged using a confocal laser scanning microscope.

### Quantitative real-time polymerase chain reaction

The PC12 cells (5 × 10^4^ cells/well) were cultured with varying concentrations of IONPs (0, 10, 20, 50 μg/ml) for 5 days. For hydrogel samples, hydrogels (250 μl) were loaded into the 24-well plate. After gelation, PC12 cells were placed at a density of 5 × 10^4^ cells per well. In all samples, total RNA was extracted after 5 days using an mRNA kit (Yeasen, China). Afterwards, 500 ng of total RNA underwent a transformation into cDNA through reverse transcription using an RT kit (Yeasen, China). Real-time PCR detection system (Thermo Fisher Scientific, USA) was utilized to measure the cycles of quantitative PCR (qPCR) using SYBR Green supermix (Yeasen, China). GAPDH (Sangon Biotech, China; Cat No: B661204) was employed as the reference gene for data normalization. The data are analyzed using the 2^−ΔΔCt^ method. The primer sequences are provided in Table 1.

**Table 1.** Sequences of primers employed in quantitative RT-PCR

Gene	Primers (F = forward, R = reverse)
NGF	F: CCAGCCTCCACCCACCTCTTC R: GCTTGCTCCTGTGAGTCCTGTTG
VEGF	F: CACGACAGAAGGGGAGCAGAAAG R: GGCACACAGGACGGCTTGAAG
GAPDH	F: GACATGCCGCCTGGAGAAAC R: AGCCCAGGATGCCCTTTAGT

### Enzyme-linked immunosorbent assay analysis

The neurotrophin levels of PC12 cells on the different hydrogels were evaluated with NGF and vascular endothelial growth factor (VEGF) enzyme-linked immunosorbent assay (ELISA) kits. After 5 days, the protein was extracted and used for the experiment according to the manufacturer’s instructions.

### The degradation and biocompatibility of hydrogel *in vivo*

The animal use protocol listed below has been reviewed and approved by the Institutional Animal Care and Use Committee (IACUC), ZJCLA (ZJCLA-IACUC-20010206). A group of 45 female Sprague Dawley (SD) rats (8 weeks old, 200–250 g) were sourced from Slac Laboratory Animal Co. For *in vivo* imaging, the 2% IONPs/fibrin hydrogel was soaked in Cyanine 5 (Cy5) fluorescent dye solution (Gene Pharma, China) for 24 h. Three SD rats for imaging and 18 SD rats for histological staining underwent anesthesia with isoflurane, followed by shaving of the dorsal hair. The dorsal skin was separated from the surrounding muscular tissue, and pure fibrin hydrogel or 2% IONPs/fibrin hydrogel containing Cy5 was subcutaneously implanted. At 1, 3, 5, 7 and 9 days postoperatively, we identified the *in vivo* fluorescence signals using the *in vivo* imaging system (IVIS) spectrum (Perkinelmer, USA) with Cy5 filtering to determine whether the signals were present or not. The rats in both groups were euthanized on Days 1, 3 and 5 postoperatively to obtain the skin from the hydrogel implantation site. The skins were stained with hematoxylin eosin (HE) for analyzing the inflammatory cell infiltration.

### Animal procedures

Twenty-four SD rats were assigned randomly to four groups (*n* = 6): autologous nerve graft (Autograft), chitosan nerve conduit containing fibrin hydrogel (Fibrin), chitosan nerve conduit containing 2% IONPs/fibrin hydrogel (2% IONPs/fibrin) and hollow chitosan nerve conduit (Hollow). The rats were administered an intraperitoneal injection of pentobarbital sodium at a concentration of 1% (w/v) and at a dosage of 40 mg/kg before the surgical procedure. The surgical procedure involved preparing the skin covering the left femur, leading to the exposure of the sciatic nerve. The Autograft group performed a suturing procedure on a 10-mm sciatic nerve segment that had been reversed. In the Fibrin and 2% IONPs/fibrin groups, the chitosan nerve conduits filled with the hydrogels were sutured with the nerve stumps in the 10-mm gaps. The Hollow group utilized an empty chitosan nerve conduit to bridge the 10-mm gap. All rats were given free access to food and water in a controlled and germ-free environment for a period of 12 weeks.

### Transmission electron microscopy and toluidine staining

At the 12-week mark following the surgical procedure, the regenerated sciatic nerves were taken out after euthanasia of the rats in each group. The specimen was subjected to a 3-h preservation period at 4°C in a 2.5% glutaraldehyde solution, post-treated with a solution of osmium tetraoxide at a concentration of 1% for a duration of 1 h, and subsequently washed, dehydrated, and embedded in Epon 812 epoxy resin. The embedded nerves were sliced into ultrathin sections with a thickness of 70 nm and semithin sections with a thickness of 700 nm. The semithin sections underwent staining with 1% toluidine blue/1% borax solution and captured using an automatic digital slide scanning system (Zeiss, Germany). For each group, we assessed the total count of myelinated axons and standardized it according to the combined area in three randomly selected fields, with the average representing myelinated axon density. The ultrathin sections were stained with lead citrate and uranyl acetate, then examined using a Tecnai G2 spirit 120 kV cryo-transmission electron microscope. TEM images were acquired from three randomly chosen fields of each sample to characterize parameters including the diameter of myelinated nerve fibers, the thickness of myelin sheaths, and the *g*-ratios based on diameter/perimeter, calculated using ImageJ software.

### Immunofluorescent staining

The sciatic nerve lesions in rat were collected from all groups (*n* = 3 per group) at 12 weeks post-operatively. 7-μm-thick transverse frozen sections were obtained from the midportion of the sciatic nerve. Immediately following blocking with 10% goat serum, the samples were incubated with anti-neural filament 200 kDa (NF200, 1:400; Sigma-Aldrich, USA) overnight at 4°C, washed three times with PBS, and then treated with goat anti-mouse IgG H&L (Alexa Fluor 488, 1:200, ab150117, Abcam) for 1 h. Finally, the sections underwent a 10-min staining with DAPI at room temperature and imaged using an automatic digital slide scanning system (Zeiss, Germany).

### Motor functional analysis

The animal subjects were subjected to experimental evaluations within a walkway of specific dimensions, measuring 60 cm in length and 10 cm in width, with a floor covered entirely in white paper. The rats underwent preoperative training to walk in the corridor. The task was given to rats to walk down the hallway while their back paws were coated in black ink, so that as they moved, they left their footprints on the paper. The footprints were recorded at weeks 3, 6, 9 and 12 following surgery. The calculation of the sciatic function index (SFI) was performed utilizing the formula [20, 32]:

SFI=109.5((ETS−NTS)NTS)−38.3((EPL−NPL)NPL)   +13.3((EIT−NIT)NIT)−8.8

In this context, the experimental measurement for toe stretch is denoted as ETS, the normal measurement as NTS, the experimental measurement for print length as EPL, the normal measurement as NPL, the experimental measurement for intermediate toe stretch as EIT and the normal measurement as NIT.

### Restoration of function in the target gastrocnemius muscle

After 12 weeks postoperatively, the rats were euthanized and the gastrocnemius muscles from both ipsilateral and contralateral sites were harvested and promptly weighed. The muscle samples were subsequently immersed in 4% paraformaldehyde at 4°C for 7 days and later transversely sectioned into paraffin sections with a thickness of 7 μm, and stained with HE and Masson’s trichrome. The images were acquired using an automatic digital slide scanning system (Zeiss, Germany) and analyzed using ImageJ software.

### Statistical analysis

All statistical data were examined using GraphPad Prism 7 and are expressed as mean ± standard deviation (SD). ImageJ 2.0 was used to analyze the images. Group comparisons were conducted using one-way analysis of variance (ANOVA). When variances were equal, Tukey’s *post-hoc* test was used. When variances were unequal, Dunnett’s T3 *post-hoc* test was used. A significance level of *P* < 0.05 was used to determine statistical significance.

## Results

### Characterization of IONPs/fibrin hydrogels

**Figure 2.**
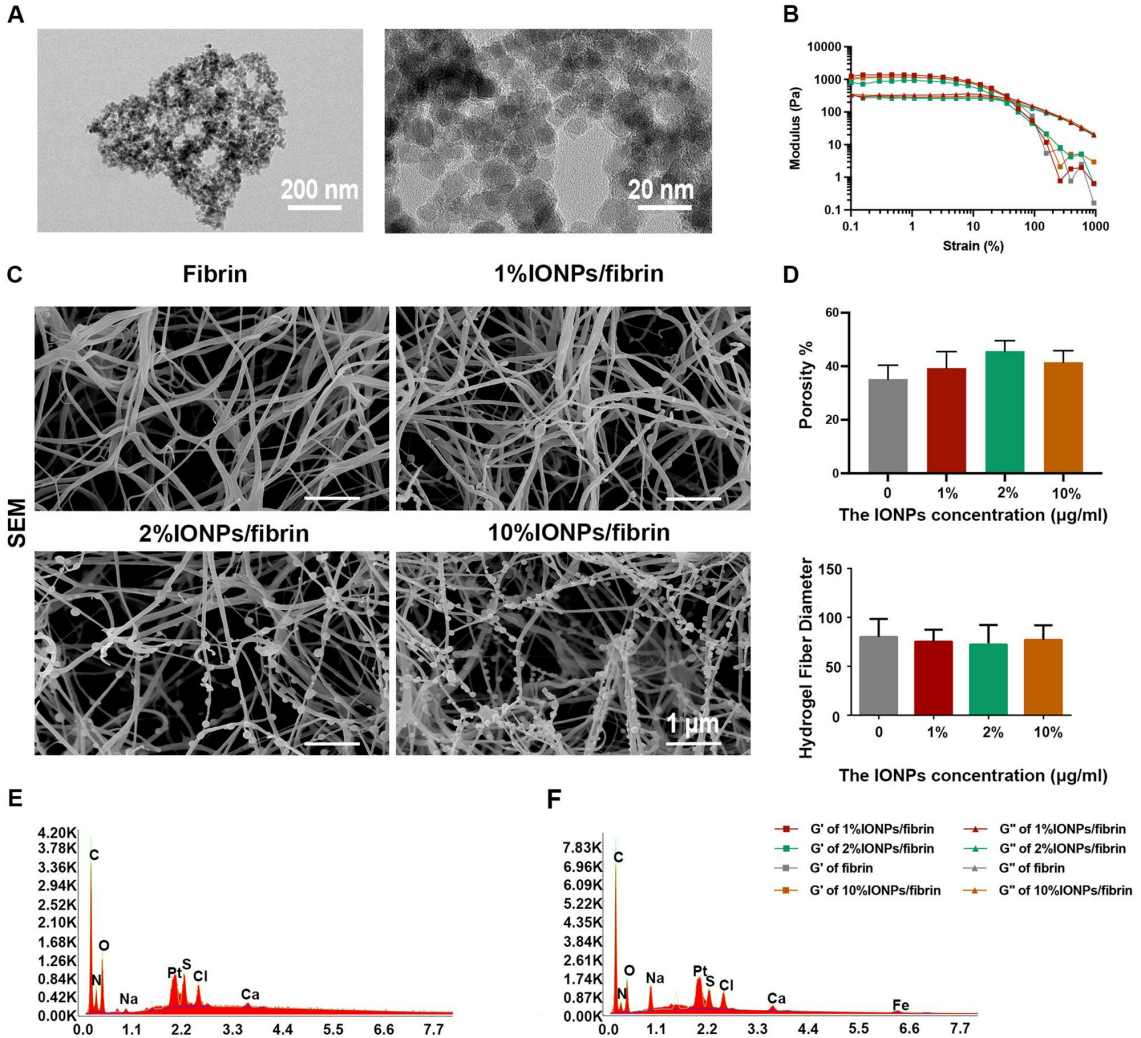
Characterization of IONPs/fibrin hydrogels. (**A**) TEM images of IONPs used in this study. (**B**) Rheological measurements of storage (*G*′) and loss (*G*′′) moduli of different hydrogels as a function of the strain (from 0.1% to 1000%) in a constant frequency (1 Hz) mode. (**C**) SEM images of the hydrogels. (**D**) The porosity and fiber diameter of the hydrogels were measured by ImageJ software based on SEM images. EDS spectrums of (**E**) fibrin hydrogel and (**F**) 2% IONPs/fibrin hydrogel.

We characterized the IONPs used in this study by using TEM. As shown in Figure 2A, the size of the IONPs was around 10∼30 nm. Utilizing an enzyme cross-linking technique, fibrin hydrogels containing different concentrations of IONPs were synthesized, enabling precise control over the formation of the hydrogels and promoting a uniform distribution of the IONPs within them. We then performed rheological measurements of the mechanical properties of the hydrogels (Figure 2B). The storage modulus (*G*′) positively correlates with the mechanical rigidity, while the loss modulus (*G*″) is related to the viscous properties of the hydrogel. In stress/strain sweep test, the *G*′ of each hydrogel remained constant when the shear strain was between 0.1% and ∼1% and decreased slowly after 1%, whereas *G*″ showed the same trend at <10%, followed by a steady decrease, indicating that the limits of the linear viscoelastic region reached at around 1% strain. The *G*′ of the hydrogels is about 1 kPa, and the *G*″ is about 300 Pa, which is consistent with our previous study. The *G*′ and *G*″ intersect at 13%, indicating that when the strain is less than 13%, the material is in a gel state. SEM was employed to analyze the microstructure of these hydrogels. The experiment revealed that fibrin hydrogels with various amounts of IONPs displayed a uniformly porous mesh structure, which is consistent with previous study (Figure 2C) [33]. The porosities of fibrin hydrogels with different concentrations (0%, 1%, 2% and 10%) of IONPs were 35.74 ± 8.10%, 39.33 ± 6.15%, 45.66 ± 3.90%, 43.18 ± 5.81%, respectively, whereas the diameters of the nanofibers in the corresponding groups were 80.00 ± 18.52 nm, 75.33 ± 12.06 nm, 72.33 ± 20.01 nm, 77.00 ± 15.1 nm, respectively (Figure 2D). These results showed that the concentrations of IONPs had no significant influence on the porosities and diameters of hydrogels. The respective sizes of fibrin hydrogel and IONPs/fibrin hydrogels with varying concentrations are consistent with previous study [34]. The EDS spectrum for fibrin hydrogel showed that the percent of Fe element was 0%, indicating no existence of IONPs, whereas the EDS spectrum for 2% IONPs/fibrin hydrogel showed that the presence of Fe element, revealing the existence of IONPs (Figure 2E and F).

The IONPs were well-distributed in the nanofibers, as reported previously [35]. The IONPs and nanofibers formed a distinctive spherical structure in SEM due to their strong interaction. The results indicated that the enzyme cross-linking method was appropriate for fabricating IONPs/fibrin hydrogels. It was noted that the aggregation of IONPs resulted in the formation of certain spherical entities larger than 100 nm. The agglomeration of IONPs occurred due to the attraction of opposite charges between nanofibers and IONPs. Based on SEM and EDS analysis, it was observed that the nanofibers effectively encapsulated the IONPs, resulting in the formation of a robust composite structure. Furthermore, our research revealed that the addition of IONPs did not have a significant impact on the general microstructure of the fibrin hydrogel. The structure of the fibrin hydrogel is predominantly influenced by the concentrations of thrombin and fibrinogen [35]. Hence, our findings suggest that nanofibers have the potential to serve as an efficient vehicle for integrating IONPs into fibrin hydrogels while maintaining their inherent microstructure.

### *In vitro* biocompatibility of IONPs/fibrin hydrogels

**Figure 3.**
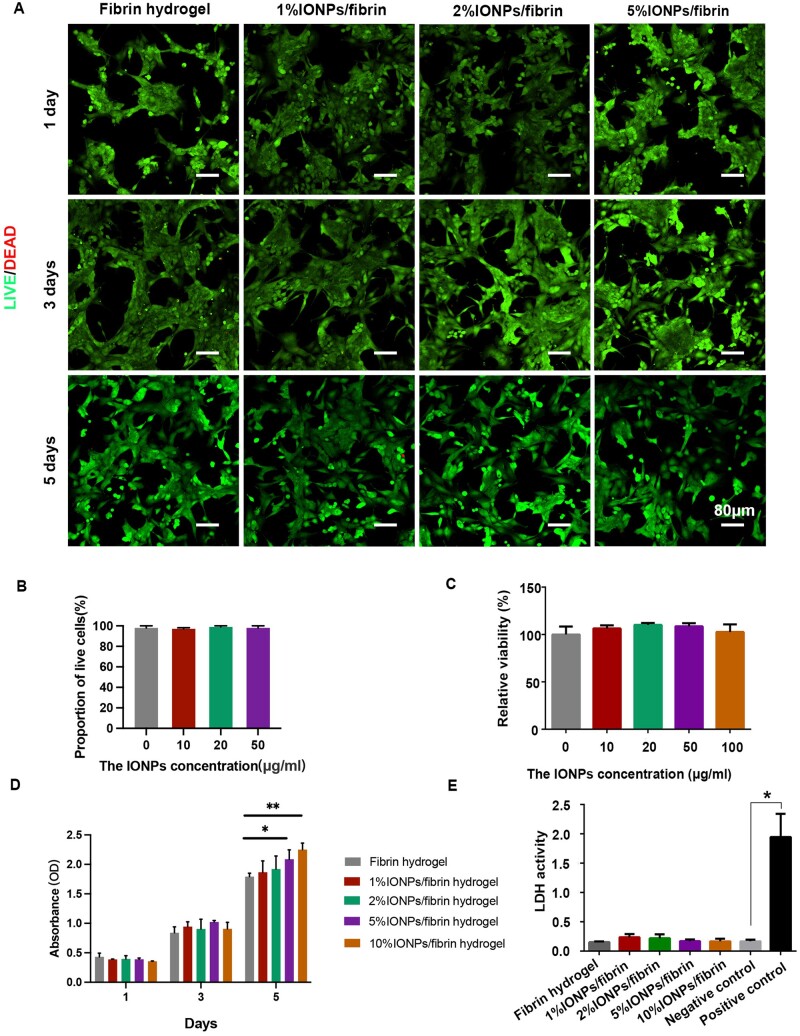
*In vitro* biocompatibility of IONPs/fibrin hydrogels. (**A**) The staining of live and dead cells at 1, 3 and 5 days after cultivation on various hydrogels. The color green represents viable cells, whereas the color red represents nonviable cells in the image. (**B**) The proportion of live cells quantified by ImageJ based on the live/dead staining images. (**C**) Cell viability of PC12 cells on hydrogels with varied concentrations of IONPs for 24 h (*n* = 3). (**D**) Cell proliferation of PC12 cells on different hydrogels analyzed via CCK8 assay (*n* = 3). (**E**) Cell membrane integrity analyzed via LDH leakage in PC12 cells incubated for 2 days (*n* = 4). *P < 0.05, and **P < 0.01.

The *in vitro* toxicity of IONPs/fibrin hydrogels was evaluated on PC12 cells. The live/dead assay revealed that live cells on IONPs/fibrin hydrogels exhibited nearly 100% viability, comparable to that on fibrin hydrogel, with no significant difference observed among the groups (*P* > 0.05) (Figure 3A and B). The presence of IONPs did not induce toxicity in PC12 cells when exposed to IONPs/fibrin hydrogels. The cell viability assay showed similar results between the IONPs/fibrin hydrogels and the fibrin hydrogel group, indicating no significant difference in cell viability (Figure 3C). Additionally, the proliferation of PC12 cells on fibrin and IONPs/fibrin hydrogels was evaluated (Figure 3D). There was no significant difference in cell proliferation rate among different groups in Day 1 and 3, while the cell proliferation rate in 5% IONPs/fibrin and 10% IONPs/fibrin groups was significantly higher than that in the fibrin group in Day 5. To further evaluate the cytotoxicity of IONPs/fibrin hydrogel, the LDH-releasing assay was conducted (Figure 3E). The untreated cells were used as the negative control. In comparison, the positive control group, consisting of cells treated with LDH release solution, exhibited a significantly higher LDH activity when compared to the negative control group, indicating a notable cellular toxicity level (*P* < 0.05). However, the LDH activities in fibrin or IONPs/fibrin groups showed no significant difference from that in the negative control group (*P* > 0.05). The results indicated no significant cytotoxicity after exposure to fibrin hydrogel or IONPs/fibrin hydrogels during incubation. Based on these findings, IONPs/fibrin hydrogel proved to be biocompatible, making it a promising candidate for nerve regeneration applications as it can be safely implanted into tissues.

### Relative gene expression in PC12 cells

**Figure 4.**
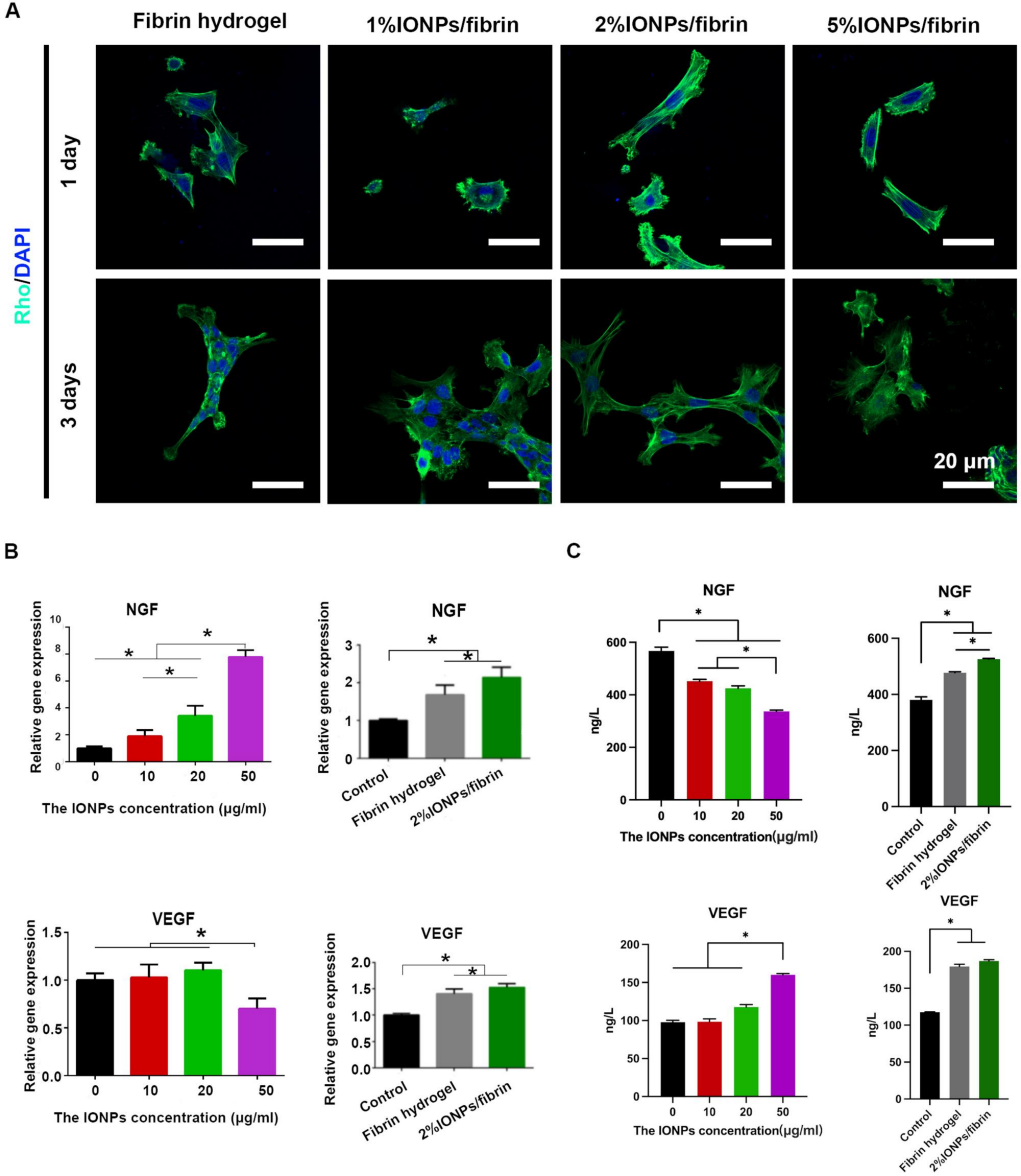
PC12 cell morphology and neurotrophin levels. (**A**) Representative confocal microscopy images of PC12 cells on different hydrogels. (**B**) Gene expression of NGF and VEGF in PC12 cells incubated with different concentrations (0, 10, 20 and 50 μg/ml) of IONPs and on hydrogels (fibrin and 2% IONPs/fibrin) for 5 days. (**C**) The levels of NGF and VEGF of PC12 cells incubated with different concentrations (0, 10, 20 and 50 μg/ml) of IONPs and on hydrogels (fibrin and 2% IONPs/fibrin) for 5 days. *P < 0.05 (*n* = 3).

The PC12 cells adhere well to the hydrogel (Figure 4A). In order to figure out the effects of IONPs alone on PC12 cells, the gene expression of PC12 cells was examined by co-culturing with different concentrations of IONPs. As shown in Figure 4B, the expression of nerve growth factor (NGF) in the groups treated with IONPs (20 and 50 μg/ml) significantly increased compared to that of control group (*P* < 0.05). Within this specific concentration range, a strong correlation is evident between the expression of the NGF gene and the concentration of IONPs. IONPs were found to exert a favorable influence on PC12 cells, whereby the level of IONP concentration played a critical role in the regulation of NGF expression. Additionally, the quantification of VEGF gene expression in PC12 cells was assessed through the co-culture with IONPs at varying concentrations. Interestingly, the relatively higher concentration of IONPs (50 μg/ml) decreased the expression of VEGF gene in PC12 cells compared to the control group (*P* < 0.05), while no significant difference was observed between the IONPs (10 and 20 μg/ml) and control groups. Additionally, we examined the levels of NGF and VEGF expression in PC12 cells during their co-cultivation with 2% IONPs/fibrin and fibrin hydrogels. It was shown that the presence of 2% IONPs/fibrin and fibrin hydrogels significantly increased the expression of the NGF gene compared to the control group (*P* < 0.05). Besides, there was significantly different between the 2% IONPs/fibrin hydrogel and fibrin hydrogel (*P* < 0.05), indicating that the NGF gene expression in PC12 cells was enhanced when IONPs were added to fibrin hydrogel. The combination of IONPs and fibrin hydrogel has been found to be a potentially effective approach in enhancing NGF gene expression in PC12 cells. In addition, the expression of the VEGF gene was found to be significantly higher in both the fibrin hydrogel and 2% IONPs/fibrin hydrogel groups when compared, demonstrating a notable difference between the two groups (*P* < 0.05), which may enhance angiogenesis [36]. The ELISA results indicate that the trend of VEGF levels in PC12 cells incubated with different concentrations and on hydrogels is consistent with the PCR results (Figure 4C). However, the levels of NGF in PC12 cells incubated with different concentrations of IONPs exhibited an opposite trend to the NGF gene expression, with higher concentrations of IONPs correlating to lower protein levels. But the NGF levels in fibrin and 2% IONPs/fibrin groups were significantly higher than that in the control group, while the NGF level in 2% IONPs/fibrin group was significantly higher than that in fibrin group. Therefore, both the combination of 2% IONPs with fibrin hydrogel and pure fibrin hydrogel have the ability to increase the expression of NGF and VEGF in PC12 cells, which is beneficial for nerve regeneration.

### *In vivo* degradation and biocompatibility of IONPs/fibrin hydrogel

**Figure 5.**
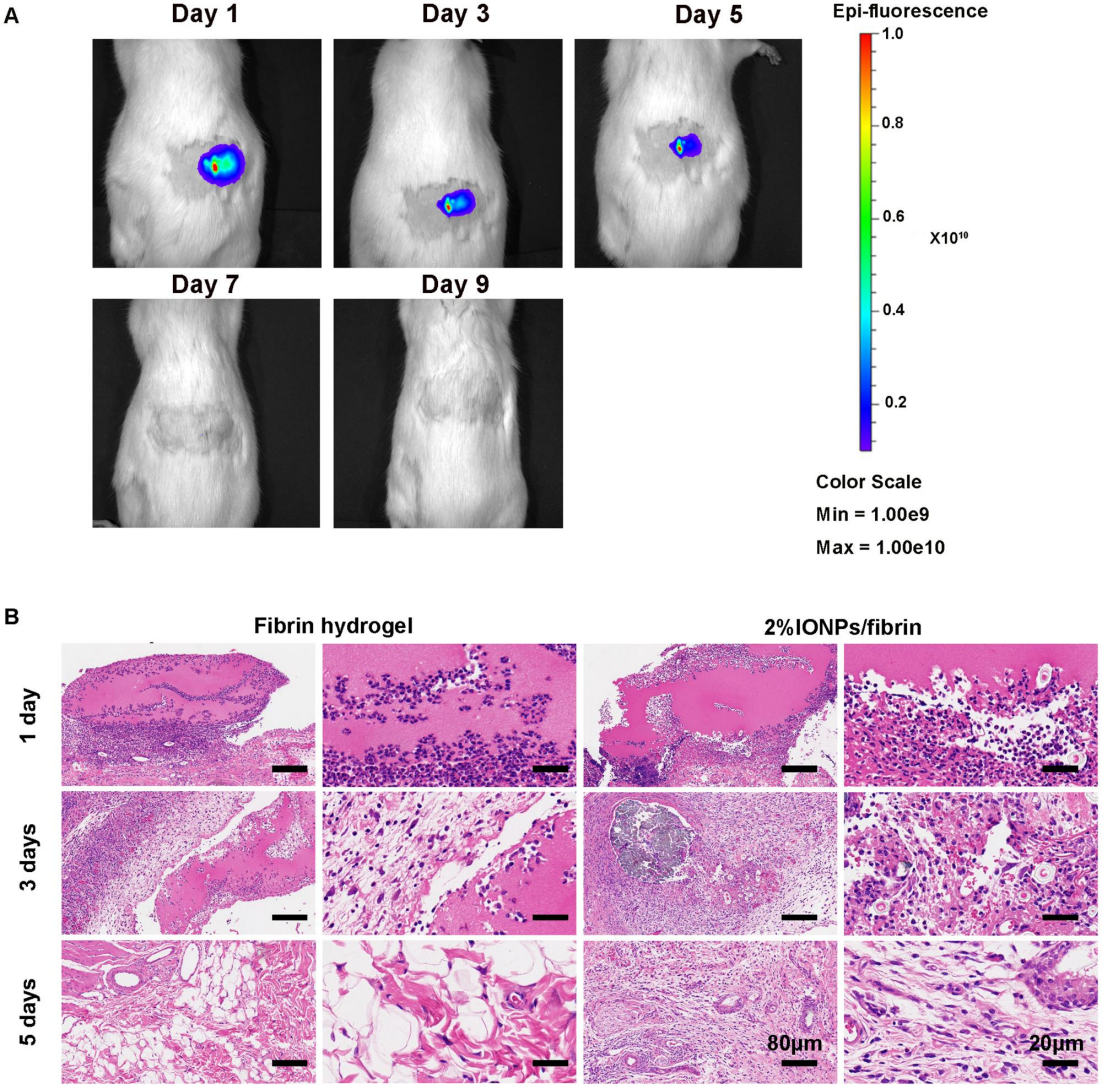
*In vivo* degradation and biocompatibility of 2% IONPs/fibrin hydrogel. (**A**) The IVIS images of Cy5-containing 2% IONPs/fibrin hydrogel implants. (**B**) HE staining of skin tissue with fibrin and 2% IONPs/fibrin hydrogel implants after 1, 3 and 5 days of implantation (*n* = 3).

The degradation properties of the hydrogels were evaluated by subcutaneously implanting 2% IONPs/fibrin hydrogels containing Cy5 in rats for a duration of 9 days (Figure 5A). The degradation process was visualized in real time with IVIS. The Cy5-labeled hydrogels served the purpose of *in vivo* visualization to comprehend degradation rates and extents, contributing to an enhanced understanding of the degradation process. The intensity of the fluorescence in the fibrin hydrogel containing 2% IONPs decreased gradually over a period from the first day to the ninth day. By Day 7, most of the fluorescence had disappeared, suggesting that the hydrogels underwent degradation. As the hydrogel broke down, the fluorophores were set free and became undetectable, leading to a reduction in the intensity of fluorescence. At 1, 3 and 5 days postoperatively, the implants and the surrounding skin were removed and stained by HE for observation (Figure 5B). At 1 day after subcutaneous implantation, both fibrin and 2% IONPs/fibrin groups of hydrogels are visible under HE staining, with a significant amount of cell infiltration at the edges. After 3 days, the volume of the hydrogels decreases, and partial degradation is observed. At 5 days, the granulation tissue shrank significantly without apparent inflammation, and the fluorescence intensity weakened as well, aligning with the IVIS findings. Overall, the *in vivo* results demonstrated that the combination of 2% IONPs with a fibrin hydrogel exhibited excellent biocompatibility and exhibited a rapid degradation rate.

### Morphology analysis of the regenerated nerves

**Figure 6.**
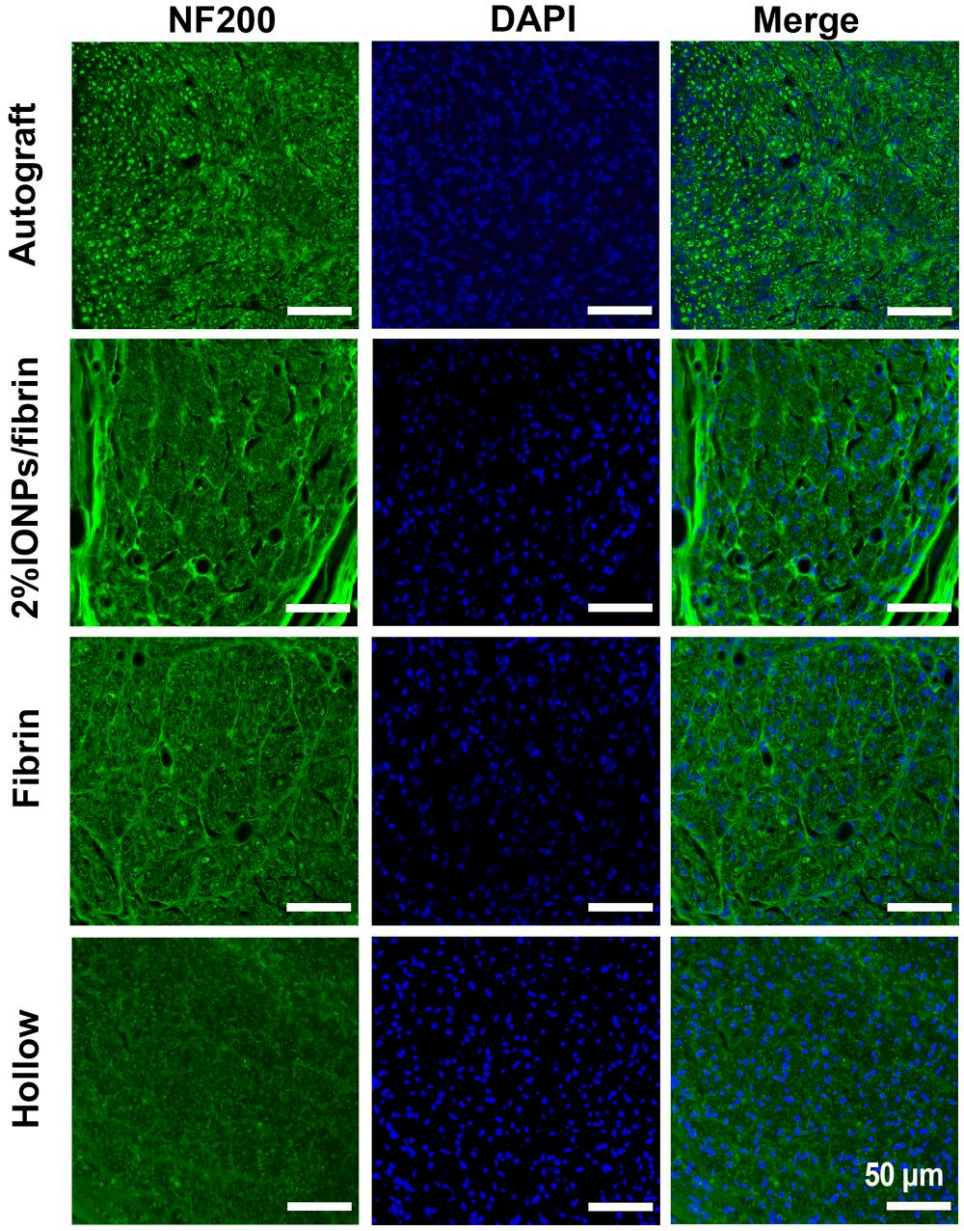
Immunofluorescence staining of regenerated nerve segments in the mid-portion at 12 weeks post-surgery. Axons and nuclei underwent staining with NF200 (green) and DAPI (blue), respectively (*n* = 3).

The implantation of four groups of nerve grafts (Autograft, 2% IONPs/fibrin, Fibrin and Hollow) was performed to connect the 10-mm-long transected gaps in sciatic nerve. To evaluate the regeneration of axons following injury across all experimental groups, immunofluorescence staining was employed to visualize the morphologies of the axons (Figure 6). There were variations in the distribution of regenerating nerves within the lesions in different groups. The hollow group displayed significantly fewer regenerating axons. Conversely, the other three groups showed a high abundance of regenerating axons especially in the autograft group. The autograft group showed the highest number of regenerating axons, suggesting a beneficial effect of the graft procedure on axonal regeneration.

**Figure 7.**
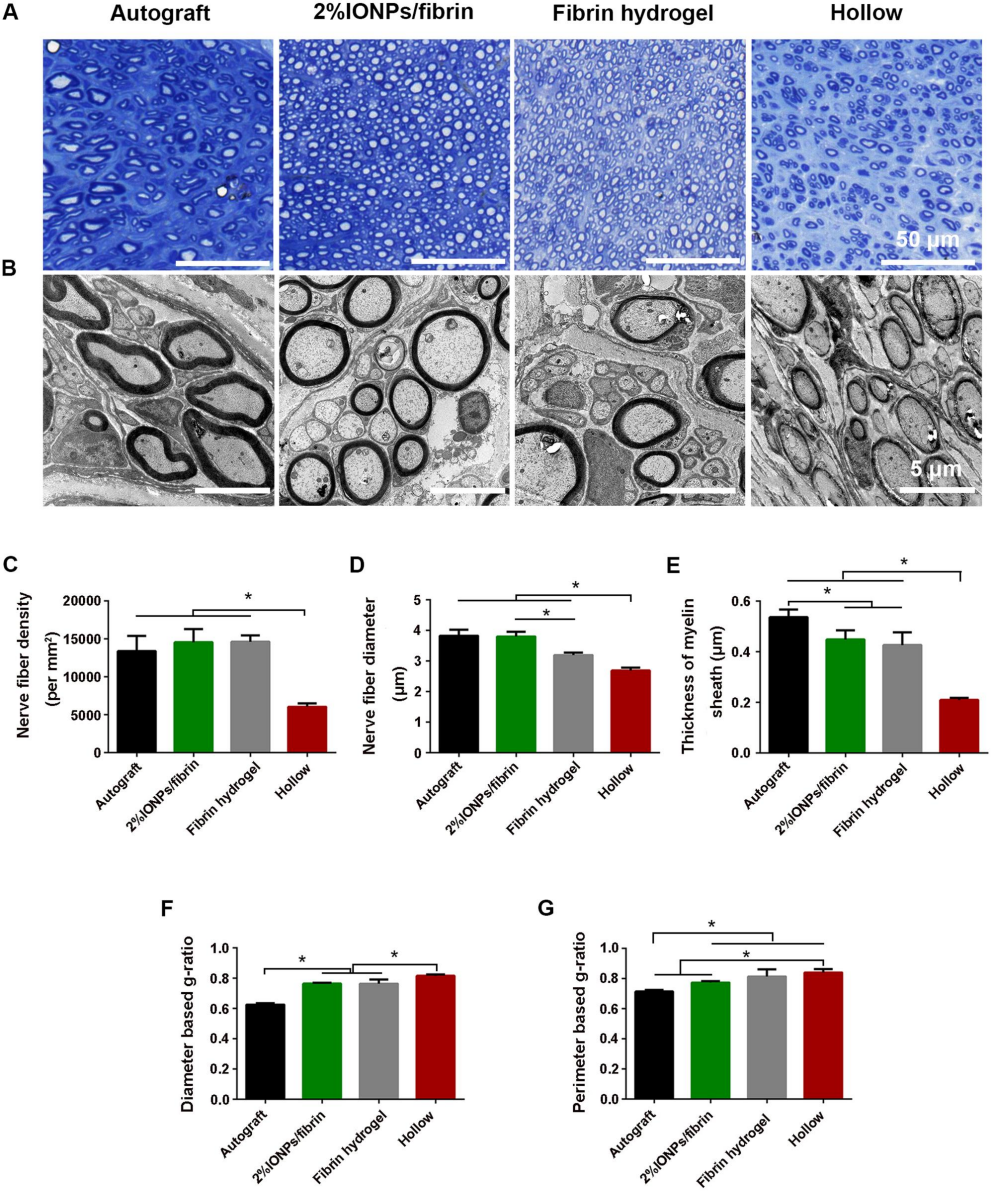
The process of nerve regrowth within the implanted region was observed 12 weeks after the surgical procedure. (**A**) Transverse sections stained with toluidine blue. (**B**) The TEM images of the regenerated axons provide valuable insight into their structure and composition. Histomorphometric analysis assesses the density of myelinated axons (**C**), the diameter of myelinated axons (**D**), the thickness of the myelin sheath (**E**), diameter-based *g*-ratio (**F**) and perimeter-based *g*-ratio (**G**) when evaluating regenerated axons. *P < 0.05 (*n* = 3).

In addition, these findings were confirmed through toluidine blue staining and TEM analysis of the intermediate sections of the regenerated nerve (Figure 7A and B). We observed the presence of well-developed myelinated axons that exhibited thick myelin sheaths within the autograft, 2% IONPs/fibrin, and fibrin groups. In contrast, the hollow group displayed myelinated axons that were both small and thin. This occurrence is probably a result of using 2% IONPs/fibrin or just fibrin, which assists in furnishing structural reinforcement to the healing nerve. Conversely, the hollow group owned fewer and less myelinated axons due to the lack of such support. The density of myelinated axons in the autograft (13 369.4 ± 2008 nerves/mm^2^), 2% IONPs/fibrin hydrogel (14 542.4 ± 1727.8 nerves/mm^2^), and fibrin hydrogel groups (14 603 ± 838.1 nerves/mm^2^) were significantly higher than that of the hollow group (6026.7 ± 451.6 nerves/mm^2^, *P* < 0.05), which suggested that both 2% IONPs/fibrin hydrogel and fibrin hydrogel created a favorable environment for axon regrowth compared to hollow nerve conduit (Figure 7C). Compared to the fibrin (3.19 ± 0.08 μm) and hollow groups (2.68 ± 0.09 μm), autograft (3.82 ± 0.2 μm) and 2% IONPs/fibrin groups (3.8 ± 0.16 μm) showed a significantly larger diameter of myelinated axons (*P* < 0.05, Figure 7D). Moreover, the diameter of the axons in the fibrin group was significantly greater than that of the hollow group (*P* < 0.05). Significant difference was not observed between the 2% IONPs/fibrin and autograft groups. The results demonstrated that the two treatments had a similar effect on the diameter of myelinated axons. The thickness of myelin sheath in 2% IONPs/fibrin (0.45 ± 0.04 μm) and fibrin (0.43 ± 0.05 μm) groups were markedly larger compared to the hollow group (0.21 ± 0.01 μm, *P* < 0.05), but were still smaller than that observed in the autograft group (0.54 ± 0.03 μm, *P* < 0.05, Figure 7E). There was no significant difference in myelin thickness between the 2% IONPs/fibrin and fibrin groups (*P* > 0.05). Afterward, *g*-ratios based on diameter/perimeter were computed to evaluate the extent of nerve fiber myelination. As noted previously, a *g*-ratio of around 0.6 is considered optimal for healthy peripheral nerves [37, 38]. The *g*-ratio based on diameter in the 2% IONPs/fibrin (0.76 ± 0.00) and fibrin (0.76 ± 0.03) groups was substantially smaller compared to the hollow group (0.82 ± 0.01, *P* < 0.05), but it was still significantly greater compared to the autograft group (0.62 ± 0.01, *P* < 0.05) (Figure 7F). This suggests that incorporating fillings with 2% IONPs/fibrin and fibrin hydrogels alone into hollow NGCs could considerably improve axon myelination, and the myelination process was not affected by the presence of IONPs. The *g*-ratio based on perimeter in the 2% IONPs/fibrin hydrogel group (0.77 ± 0.01) was considerably smaller than that in the hollow groups (0.83 ± 0.01, *P* < 0.05), but it was still notably larger compared to the autograft group (0.71 ± 0.01, *P* < 0.05) (Figure 7G). Altogether, our findings offered proof demonstrating the favorable impact of using 2% IONPs/fibrin hydrogel on the growth of axons and the development of myelin.

### Restoration of function in the target gastrocnemius muscle

**Figure 8.**
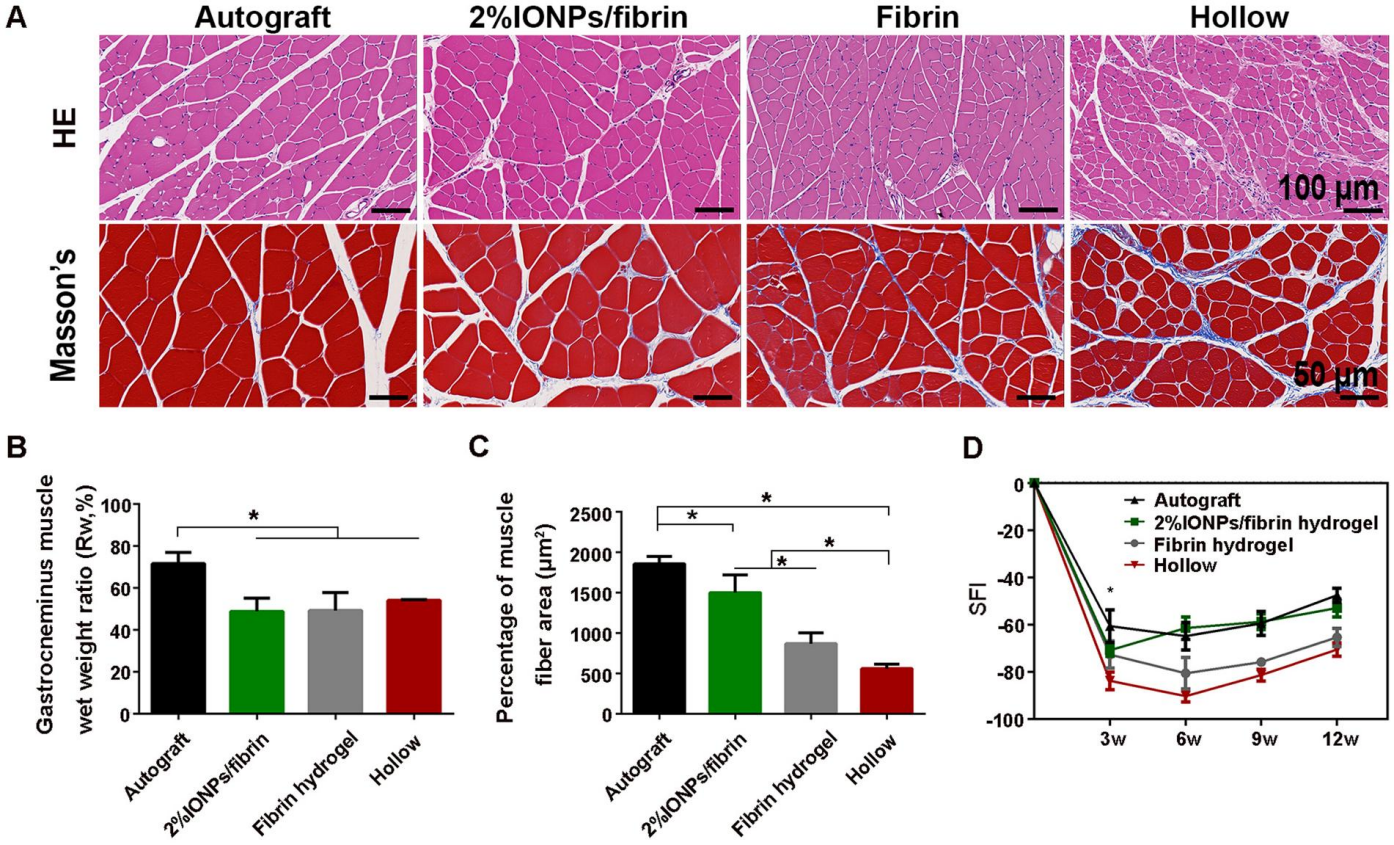
Restoration of function in the target gastrocnemius muscle. (**A**) HE staining and Masson’s trichrome staining of cross-sectional muscles of the ipsilateral side. (**B**) Statistical analysis of muscle wet weight ratio comparing the ipsilateral side to the contralateral side. (**C**) The average cross-sectional areas of the muscle fibers observed in Masson’s trichrome staining. (**D**) SFI values in 3, 6, 9 and 12 weeks after surgery. *P < 0.05 (*n* = 3).

The re-innervation process in target muscles is essential for the restoration of function after nerve injury. Twelve weeks post-surgery, the gastrocnemius muscles in all groups were collected, weighed and stained with HE and Masson’s trichrome (Figure 8A). In order to assess the muscle atrophy in a quantitative manner, the muscle weight ratio was determined by comparing the muscle weight on the ipsilateral side to that on the contralateral side (Figure 8B). There was no statistically significant difference in the muscle wet weight ratio between 2% IONPs/fibrin hydrogel (48.86% ± 6.34%), Fibrin hydrogel (49.28% ± 8.58%) and Hollow groups (54.09% ± 0.48%), but were statistically lower than that of Autograft group (71.64% ± 5.3%) (*P* < 0.05). Furthermore, the average cross-sectional area of gastrocnemius muscle fibers in the 2% IONPs/fibrin hydrogel group (1498.84 ± 220.53 μm^2^) closely resembled that in the Autograft group (1855.79 ± 91.89 μm^2^, *P* > 0.05), but notably greater than those in the Fibrin (868.6 ± 132.37 μm^2^, *P* < 0.05) and Hollow groups (559.52 ± 54.54 μm^2^, *P* < 0.05) (Figure 8C). Based on the staining results, there was an increasing amount of collagen fibrous tissues (blue in the images) in fibrin hydrogel and hollow groups. These observations could be related to the presence of higher amounts of collagen fibrous tissues surrounding the muscle fibers. These results imply that muscle re-innervation was enhanced in the 2% IONPs/fibrin hydrogel and Autograft groups.

To assess restoration of motor function during sciatic nerve regeneration, walking track analysis was conducted every 3 weeks post-operatively. The SFI was computed based on the footprints in order to analysis sciatic nerve function [30]. SFI scores nearing 0 indicate typical motor function, while scores nearing −100 indicate complete dysfunction. The SFI values diminished in all groups 3 weeks post-operatively, which indicates muscle denervation and impaired motor function in the early stage (Figure 8D). This finding is consistent with previous study [29]. Between 3 and 12 weeks, SFI values of the 2% IONPs/fibrin hydrogel group showed a gradual increase, indicating that successful nerve regeneration led to the restoration of muscle innervation and recovery. However, SFI values of the other groups demonstrated a rising trend from 6 to 12 weeks, as previously reported [29]. At 3 weeks, the SFI values of the group treated with 2% IONPs/fibrin hydrogel and the group treated with fibrin hydrogel alone were significantly higher compared to the hollow group (*P* < 0.05), but significantly lower than the autograft group (*P* < 0.05). Between the 6th and 12th week, there were no significant differences observed in the SFI values of the 2% IONPs/fibrin hydrogel and autograft groups (*P* > 0.05), indicating that the combination of 2% IONPs and fibrin hydrogel could potentially enhance the restoration of motor function, implies a favorable influence on recovery.

## Discussion

PNI continues to present a difficult situation from a medical perspective, frequently leading to prolonged impairment. Although autologous nerve transplantation is considered the benchmark, it does have certain limitations. The rise in popularity of magnetic scaffolds in tissue regeneration, driven by their advantageous physical and biological characteristics as mentioned previously, motivated our investigation. As a result, we synthesized IONPs/fibrin hydrogels and evaluated their compatibility with living tissues as well as their effect on the regeneration of peripheral nerves. The findings unveiled that IONPs/fibrin hydrogels displayed outstanding biocompatibility, stimulating the growth of nerve projections, and aiding in the restoration of function *in vivo*.

Here, IONPs were well distributed in nanofiber which indicated fibrin hydrogel has the ability for encapsulating IONPs. The nanostructure of IONPs/fibrin hydrogel was revealed to exhibit a porous architecture closely resembling that of the ECM. As reported previously, a porous framework holds a crucial position in promoting the development and movement of cells, with the fibers ideally smaller than the cells [39]. Here, the nanostructure of the IONPs/fibrin hydrogel proves to be advantageous for cells growth. Following incubation with PC12 cells, it was demonstrated that the incorporation of IONPs in fibrin hydrogel exhibited favorable biocompatibility at a concentration 100 μg/ml. Besides, the integrity of the cell membrane in PC12 cells was not affected as well. Notably, a recent study spotlighting magnetic-responsive aligned fibrin hydrogel in spinal cord regeneration has been reported [11]. The viability of PC12 cells co-cultured with IONPs (lower than 200 μg/ml) were over 90%, and the proliferation of PC12 cells in magnetic-responsive aligned fibrin hydrogel and aligned fibrin hydrogel had no significant difference. Hence, the IONPs/fibrin hydrogel employed in this research exhibited no harmful effects on cells and maintained the integrity of the cell membrane. Previous studies have shown that IONPs possess unique properties that can enhance cell proliferation. These nanoparticles have been reported to promote cellular proliferation through various mechanisms, including the activation of cell signaling pathways involved in cell growth and division, modulation of cellular adhesion and migration and stimulation of cellular metabolic activity. Additionally, the fibrin hydrogel itself serves as a biocompatible and supportive matrix for cell growth and proliferation. The 3-dimensional structure of the hydrogel provides a conducive environment for cell adhesion, spreading and proliferation. Furthermore, the release of growth factors or other bioactive molecules from the hydrogel may also contribute to the observed increase in cell proliferation. Overall, the observed promotion of PC12 cell proliferation by the IONPs/fibrin hydrogel in Figure 3D suggests that this biomaterial composite has the potential to support cell growth and regeneration, which is promising for applications in tissue engineering and regenerative medicine. Further studies are warranted to elucidate the specific mechanisms underlying this effect and to optimize the design of the IONPs/fibrin hydrogel for various biomedical applications.

Moreover, effective nerve regeneration cannot be achieved without the degradation of the hydrogel. In general, the degradation of synthetic polymer materials tends to be slower than the degradation of natural biological materials. For example, the degradation duration for polylactic acid and polyglycolide is a matter of weeks, while polycaprolactone can take as long as 2 years to degrade [40]. Here, the degradation of 2% IONPs/fibrin hydrogel took approximately 7 days, which is similar with the degradation time of fibrin hydrogel (1 week) [41]. The degradation time of the IONPs/fibrin hydrogel is suitable for promoting nerve growth in nerve guide conduits, progressing at a rate of 1 mm per day [29]. Additionally, the hydrogel facilitated axonal growth during the initial stages of regeneration. This early enhancement in axonal growth holds the potential to expedite target muscle reinnervation in later stages, thereby accelerating the overall regeneration process. In the meantime, the *in vivo* experiment demonstrated favorable biocompatibility of the hydrogel comprising 2% IONPs and fibrin. The potential connection to the anti-inflammatory properties of fibrin hydrogel and its ability to attract anti-inflammatory macrophages has been hinted at in prior research [31].

In order to evaluate the effect of 2% IONPs/fibrin hydrogel on sciatic nerve regeneration, the examination encompassed the histomorphometric characteristics of the sciatic nerve and gastrocnemius muscle, the gastrocnemius muscle’s weight ratio and the motor function. The use of 2% IONPs mixed with fibrin hydrogel led to a notable increase in the growth of axons, particularly in terms of their diameter, while having no impact on the formation of myelin around the regenerated nerve fibers. The density of myelinated axons in the hollow group was significantly lower than that observed in the other groups, suggesting limited nerve regeneration potential. The lack of sufficient physical support from hydrogels in the hollow group could be the cause of this. In other words, the porous structure of the 2% IONPs/fibrin and fibrin hydrogels promoted the elongation of axons. In terms of nerve development, the size of the nerves and the thickness of their protective coating, called the myelin sheath, are essential factors. Initially, when nerves start to heal, there is typically an inclination for both the size of the nerve and the thickness of its protective myelin sheath to grow, ultimately resulting in a decrease of the *g*-ratio towards 0.6 [42]. Nerve maturity is often indicated by a larger diameter, whereas regenerated nerves tend to have a smaller diameter [43]. The size of regrown nerves in the 2% IONPs/fibrin group exhibited a notable increase in comparison to the fibrin group, similar to the autograft group. This suggests that the use of 2% IONPs/fibrin hydrogel can effectively enhance the process of nerve regeneration. The difference in myelin sheath thickness between the groups that received 2% IONPs/fibrin and fibrin hydrogel was not statistically significant, suggesting that the inclusion of 2% IONPs in fibrin hydrogel did not impact the regeneration of nerves. The *g*-ratio based on the perimeter in the 2% IONPs/fibrin and fibrin hydrogel groups showed a difference, which could possibly be explained by the variation in diameter among these groups. Nevertheless, despite the delay in nerve maturity observed in the fibrin group, it exhibited superior performance in comparison to the hollow group in terms of other parameters. The sciatic nerve injury caused denervation atrophy in the gastrocnemius muscles. The speed at which axons regenerate determines the extent of re-innervation in the respective muscles. At the initial phase following the surgical procedure, the recovery of motor function in the 2% IONPs/fibrin hydrogel group showed similarity to that of the autograft group. According to a previous report, the initiation of axonal bridging in 10-mm defects using hollow nerve conduits occurs 3 weeks after injury [44]. Motor function recovery was observed to begin at 3 weeks after the injury, suggesting that utilizing a combination of 2% IONPs/fibrin hydrogel can effectively improve functional rehabilitation following a sciatic nerve injury. The findings suggest that the cross-sectional area of large myelinated axons and muscle fibers in the 2% IONPs/fibrin hydrogel group is comparable to that in the autograft group, as evidenced by the results in SFI, pointing to agreement. Moreover, the investigation conducted on SD rats demonstrated that the 2% implantation of IONPs/fibrin hydrogel underwent degradation within a span of 7 days without displaying any visible signs of inflammation. Overall, the efficacy of utilizing 2% IONPs along with a fibrin hydrogel for repairing the sciatic nerve surpassed that of using solely a fibrin hydrogel or a hollow conduit.

Notably, 2% IONPs incorporated into a fibrin hydrogel led to elevated levels of NGF and VEGF in PC12 cells, consistent with the *in vivo* results. As the recent study reported, IONPs at a concentration of 200 μg/ml have a greater effect in inducing the expression of the NGF gene in PC12 cells compared to the control group [11]. This suggests that, firstly, the production and secretion of NGF could be increased by incorporating 2% IONPs/fibrin hydrogel, leading to enhanced axonal outgrowth. Secondly, angiogenesis is crucial for peripheral nerve regeneration, and as reported previously, fibrin hydrogel promotes angiogenesis and enhances neurite elongation [36, 41]. About 2% IONPs incorporated into a fibrin hydrogel have the potential to improve the regeneration of peripheral nerves by facilitating the combined processes of angiogenesis and neurogenesis. As mentioned earlier, magnetic scaffolds have the capability to enhance cell proliferation and differentiation. For instance, it was noted that olfactory ecto-mesenchymal stem cells (OE-MSCs), which are a specific type of stem cells located in the nose, showed the ability to transform into cells of the nervous system when placed in the alginate magnetic scaffolds [45]. Zhang *et al.* [46] found that magnetic chitosan hydrogel-mediated neural stem cell differentiation was associated with protein corona formed on the hydrogel. It was indicated that protein corona on the surface of magnetic chitosan hydrogels significantly increased the levels of neuronal differentiation-related proteins compared to the chitosan hydrogels. Despite this, a deeper understanding of the underlying mechanisms is needed to fully harness their regenerative capabilities. The potential enhancements need to be identified and implemented, such as nanofibers that are arranged in a specific pattern and the application of magnetic fields for stimulation purposes. Additionally, a comprehensive investigation is still required to understand the underlying mechanisms in the future.

## Conclusion

In this study, various formulations of IONPs/fibrin hydrogels were developed to facilitate peripheral nerve regeneration. The 2% IONPs mixed with fibrin hydrogel demonstrated exceptional compatibility both *in vitro* and *in vivo*, leading to increased production of NGF and VEGF in PC12 cells. Furthermore, targeted application of IONPs/fibrin hydrogel effectively promoted nerve fiber regrowth and accelerated motor function restoration in a rat model with a 10-mm-long sciatic nerve injury. Consequently, the biocompatible nature of 2% IONPs/fibrin hydrogel renders it highly suitable for future applications in peripheral nerve regeneration, particularly when combined with an external magnetic field.
